# Expert panel to identify applications of virtual reality (VR) in cognitive behavioral therapy for alcohol use disorder: A Delphi study

**DOI:** 10.21203/rs.3.rs-10517029/v1

**Published:** 2026-08-06

**Authors:** Tyler Blake Wray, Molly Magill, Amy C. Traylor, Peter M. Monti

**Affiliations:** Brown University; Brown University; University of Alabama System; Brown University

**Keywords:** virtual reality, alcohol use disorder, coping skills, Delphi method, expert consensus, implementation

## Abstract

**Background.:**

Cognitive behavior therapy (CBT) is efficacious for alcohol use disorder (AUD). Virtual reality could augment CBT by immersing patients in relevant situations, scenarios, or exercises. We sought expert consensus on the most promising applications of VR and how, when, and where it might best be used.

**Methods.:**

We conducted a Delphi study with 10 experts in CBT for AUD. Round 1’s open-ended questions across 10 topics generated 89 items. In later rounds, panelists rated each item’s benefit/importance from 1 to 5. Consensus required at least 70% agreement. Items not achieving consensus were re-assessed in three subsequent survey rounds.

**Results.:**

The panel reached consensus on 84 of 89 (94%) items after three rounds. Panelists endorsed VR for practicing high-risk situations, drink refusal and assertiveness skills, and trigger identification, with therapists coaching in real time. They agreed on recommendations for introducing and debriefing and agreed VR was suitable for all levels of care. Top implementation barriers were therapist reluctance, low technology confidence, training time, cost, technical support, and reimbursement.

**Conclusions.:**

Findings offer a blueprint for using VR in CBT for AUD, including applications, delivery, timing, and setting, and a path to evaluating whether it improves outcomes beyond CBT alone.

## Introduction

Alcohol use disorder (AUD) is among the top causes of disease burden in the United States (US) (Degenhardt et al., 2018; Pan American Health Organization, 2021). This burden is sustained in part by the modest effects of available treatments, given that only about half of people with AUD achieve remission, typically only after many years (Fleury et al., 2016). Research exploring ways of improving treatment outcomes remains a high priority for the field (National Institute on Alcohol Abuse and Alcoholism, 2025).

Cognitive behavioral therapy (CBT) is an efficacious and widely practiced approach to treating AUD (Magill et al., 2019), and many counselors identify with its framework and techniques (Substance Abuse and Mental Health Services Administration, 2019). CBT for AUD comprises a range of techniques, including functional analysis, identifying high-risk situations and triggers, cognitive restructuring, and coping skills training, that aim to help patients recognize the triggers and situations that drive their drinking and how to respond with healthier coping responses (Epstein & McCrady, 2009). Many of these techniques and skills are important components of other efficacious treatment approaches, as well, such as relapse prevention and the community reinforcement approach (Hunt & Azrin, 1973; Marlatt & Donovan, 2005). Across approaches, these techniques are delivered solely through verbal discussion and written exercises. While effective, this format could feel routine and unmemorable, and it offers patients few chances to practice their coping skills in situations that resemble the ones they will face in the real world. A tool that could immerse patients in a realistic version of the situations they struggle with could make these techniques more vivid and more engaging, consistent with evidence that the quality of the coping skills patients acquire predicts better drinking outcomes more so than the number (Kiluk et al., 2010).

Virtual reality (VR) refers to computer-generated experiences that give users a convincing sense of being immersed in a digital environment (Slater & Wilbur, 1997). It achieves this subjective sense of presence by approximating the way humans naturally explore their surroundings. That is, the visual display pans with head movement and sounds are spatialized to match the user’s position and movement. VR has been used to enhance psychotherapy for a range of conditions (Bell et al., 2024; Emmelkamp & Meyerbröker, 2021; Rowland et al., 2022), but is especially well-supported for treating anxiety disorders and posttraumatic stress disorder (Carl et al., 2019a). In these applications, VR is used to facilitate exposures, which involves immersing patients in environments that encourage them to gradually confront feared stimuli or situations in order to help them learn that they are safe and tolerable (Craske et al., 2014; Rauch et al., 2012). Meta-analyses have shown that VR-assisted exposures are as effective as *in vivo* exposures for anxiety disorders (Carl et al., 2019a; Deng et al., 2019; Horigome et al., 2020). In addictions, most VR research has focused on environments that similarly facilitate exposure to alcohol or drug-related cues, such as restaurants or house parties, primarily to induce and study craving (S. H. Lee et al., 2009; Mazza et al., 2021; Simon et al., 2020). A handful of small, pilot trials have shown that using experiences like these in VR-assisted cue exposure therapy may reduce craving (Ghiţă et al., 2025; Huang et al., 2025; Ji et al., 2023; J.-H. Lee et al., 2007; Zhang et al., 2023), but very few have explored their impact on alcohol use or problems. Unlike exposure therapy for anxiety, cue exposure therapy for addiction also lacks a convincing evidence base, with meta-analyses finding little support for its efficacy in reducing drinking or preventing relapse (Conklin & Tiffany, 2002). Small pilot studies have also explored the potential utility of VR-assisted mindfulness training for addiction (Garland et al., 2024; Greenwald et al., 2024; Hargett et al., 2022; Van Doren et al., 2024), but no large trials have established clear benefit of a particular application of VR in addiction treatment.

Realizing VR’s potential in AUD treatment depends on identifying applications that would genuinely benefit from giving patients a realistic sense of presence in a relevant situation and augmenting techniques that contribute to treatment efficacy. It also depends on developing clear guidance about when and how therapists should use VR with patients and where it fits best in care. The slow uptake of VR exposure therapy for anxiety, despite strong evidence supporting it, has been attributed in part to the absence of clear guidance on integrating VR into established treatment (Wray et al., 2023). An important step toward realizing VR’s potential is consulting experts in CBT for AUD about which applications they find most promising and how VR should be used in practice.

In this study, we used the Delphi method to develop consensus about (1) promising applications of VR in AUD treatment, (2) how, when, and where VR might be used to support those applications, and (3) key barriers to implementing VR in AUD treatment settings among a panel of internationally-recognized experts (*N*=10) in CBT for AUD. We focused specifically on CBT for AUD because this approach is efficacious and a large percentage of practicing counselors identify with it (Magill et al., 2019; Substance Abuse and Mental Health Services Administration, 2019). The Delphi method is an iterative, survey-based approach for developing consensus among a group of experts about questions that lack a clear empirical answer but for which sufficient expertise exists (Hasson et al., 2000). It has been widely used to define the components of psychosocial treatments and identify ways to adapt them to various populations (de Manincor et al., 2015; King et al., 2021; Michie et al., 2013).

## Methods

### Participants

We recruited a panel of 10 experts in CBT for AUD through the authors’ professional networks, targeting recognized authorities in the field, including authors of widely used treatment manuals on the topic and principal investigators of CBT trials for SUD treatment.

### Briefing Materials and Compensation

Before Round 1, panelists received briefing materials describing the rationale and goals of the work, a brief educational video that described modern VR systems and how it has been used in treating other mental health conditions. A copy of the CBT treatment manual used as the model for this project was also provided (Epstein & McCrady, 2009), as well as a meta-analysis that compared the effects of VR-assisted exposures versus in vivo exposures (Carl et al., 2019b). Panelists were asked to assume that VR-assisted techniques would utilize a fully portable, affordable (approximately $500) headset that patients use in a seated position. VR experiences would be used exclusively during face-to-face sessions under the active guidance of a therapist who could engage patients during and after immersion and could select and customize experiences through a therapist dashboard.

### Procedures

#### Round 1: Free-text response items.

In Round 1, we sent participants an online survey that asked them to type their responses to 10 open-ended questions. These questions asked about which CBT sessions, exercises, techniques, skills, or tasks they thought might benefit from immersion, which types of environments might be helpful for augmenting these skills, what characteristics of each experience counselors should be able to customize or control, among others (see Supplementary Table 1 for a full list). After all responses were submitted, the lead author reviewed them all for uniqueness, clarity, and relevance. These responses were then used to create a quantitative survey that asked panelists to rate each response they wrote for each of the 10 questions. Items were re-phrased when necessary, but original wording was preserved whenever possible. Only a few responses were ultimately combined because of redundancy. Two co-authors independently reviewed the Round 1 responses and the Round 2 item set and agreed that it covered the included items well and captured unique content. This process yielded 89 total items to be rated in subsequent rounds across the 10 general questions. Each item consisted of a possible response to the general question being asked. For example, in response to the general question “how much you believe each technique, skill, exercise, clinical task, or session would benefit from incorporating VR?,” “relaxation training or conditioning” and “Practicing drink refusal skills, assertive communication” were items that participants were asked to rate in subsequent rounds. These items were potential applications identified by panelists in the Round 1 open-ended survey.

#### Rounds 2–4 : Ratings and Consensus.

In Round 2, panelists were asked to rate each item on a 1 (*not at all*) to 5 (*extremely*) scale, but anchors were tailored to each question. For example, questions about potential applications of VR were rated from “would benefit not at all” to “would benefit a great deal”; experience types, customization variables, therapist actions, and introduction strategies from “would not be helpful at all” to “would be extremely helpful.” Each rating question also offered an optional free-text entry box for comments or additions. In Rounds 3–4, panelists were shown the aggregated results of panelists’ responses for every item before re-rating the unresolved items, in order to encourage participants to re-consider their responses and arrive at some consensus.

Institutional review board review of this research was not required because participants were considered key informants, as they were solely asked to provide their professional opinions about the topic. Panelists were compensated $750 for participating.

### Analysis

For analysis, each item was recoded into three categories: ratings of 4 or 5 reflected agreement that an item was beneficial or important (“yes”), ratings of 1 or 2 reflected disagreement (“no”), and ratings of 3 were treated as “not sure.” We then calculated the frequency of each category for each item. Consensus was achieved when 70% of panelists (7 of 10) rated an item either “yes,” meaning the item was beneficial or important, or “no,” meaning consensus was reached that the item was *not* important. Items meeting either criterion were retired from subsequent rounds. Items meeting neither were re-presented in the next round to give panelists the opportunity to review the group’s responses in the previous round and reconsider theirs. Percentage-agreement criteria for determining consensus is common in Delphi studies using Likert scales (von der Gracht, 2012). For four questions (Q2, Q4, Q6, and Q8), consensus on a subset of options answered the overall question before every option reached threshold, either because the resolved options were mutually exclusive with the remainder or because the agreed-upon options were sufficient to answer the question. For example, once panelists agreed that the middle of a session was the optimal time to use VR, consensus on whether the beginning and end were optimal was unnecessary because those options are mutually exclusive. We therefore considered these questions complete and did not re-present their remaining items. We planned to continue until consensus was reached on all items or five rounds were completed, after which remaining items would be considered controversial. However, because we reached consensus on all but a few items after four rounds, we elected to end the study after Round 4. We used descriptive statistics for all analyses. For each item and round we report the count and percentage of panelists in each category. All analyses were conducted in Stata 16 SE (College Station, TX).

## Results

### Panel Characteristics and Participation

Panel characteristics are summarized in Table 1. All participants held doctoral degrees and were psychologists. The panel included full, associate, and assistant professors, many of whom also served in clinical and leadership roles at academic and Veteran’s Affairs medical centers. The panel was also scientifically accomplished, with a mean h-index of 41.8. There was no attrition. All 10 panelists completed all four rounds, with occasional item-level missingness.

After all rounds, the panel reached consensus on 84 of 89 items assessed (94%), with 11 resolved because items that had achieved consensus for that question made the remaining items moot. The panel reached consensus on 39 items in Round 2, 16 in Round 3, and 23 in Round 4. Sixty-one of the items assessed achieved consensus that a given item was beneficial or important, and 17 achieved consensus the item was not. Consensus results are summarized by question in Table 2, and full item-level ratings appear in Supplementary Table S1.

#### Question 1: Which CBT techniques, skills, or exercises would benefit most from incorporating VR?

Panelists endorsed VR most strongly for learning and practicing skills and exposure. Panelists agreed that VR would benefit practicing drink-refusal and assertive communication skills, learning and practicing how to manage high-risk situations, identifying triggers, anticipating future high-risk situations, and simulating the impact of seemingly irrelevant decisions on relapse risk. Participants also endorsed relaxation training. As Participant #10 noted in their comments: “*To me, the ability to safely explore high risk situations is potentially huge, as is the ability to practice relaxation or other coping skills in the face of high risk situations. A number of these techniques, skills, or exercises could be really impactful to any one client or patient -- the key is the degree to which VR would make it even* *more* *impactful*.” Equally informative were the applications that panelists agreed were *unlikely* to benefit from incorporating VR. Panelists saw limited utility in using VR to help assess drinking more accurately, to illustrate the consequences of intoxication, or to facilitate self-monitoring and logging of alcohol use over time. Whether VR could help patients identify or simulate substance-free alternative activities was controversial and did not reach consensus.

#### Questions 2 and 3: What types of VR environments would be useful to have and what should therapists be able to customize?

The panel agreed that, to support the applications of VR they identified in Question 1, environments like social situations and stressful scenarios might be most helpful to have available to counselors. Within those environments, panelists also agreed that essentially all of the factors they described in the Round 1 survey would be useful for counselors to be able to customize or tailor. However, several factors achieved consensus in early rounds of surveys, which could suggest more decisive agreement. In the first survey round (Round 2), panelists arrived at consensus that a VR platform should allow counselors to immerse patients in a variety of different types of drinking environments (e.g., restaurants, house parties, cookouts), and that counselors should be able to customize the level of “difficulty” or intensity a situation has and the level of control users have over how much they are exposed to alcohol cues. Being able to customize the types of alcohol depicted, and some demographics of the other people in an environment (age, race/ethnicity) achieved consensus in Round 3. Participant #10 captured this idea in their comments on the Round 2 rating survey:

“I feel like in the spirit of meeting people where they are and providing personally tailored content, it would matter most to the client/patient what they want! No different than a video game that lets you set difficulty, adjust conditions, etc.…play the game you feel you’re ready to play!”

A topic that arose frequently in panelists’ post-rating comments about customization was that they believed any tailoring that made the experiences look and feel more like real situations would be valuable. For example, as participant #6 said: “*I think the closer you can get a scenario to be similar to what they will actually experience and things that impact them (i.e., if loud crowded spaces seem to trigger a patient), being able to create a scenario that matches those situations as best as possible would be ideal*.” A few also mentioned that introducing some element of unpredictability into scenarios could be key to achieving more realism and capturing the real value of VR. For instance, participant #10 noted: “*I like the idea of unpredictability -- this gives a safe context in which unexpected high risk situations could be introduced and ‘what would I do if this happened in the real world?’ could be explored*.”

#### Questions 4–8: How should VR be used in sessions, and what settings might be ideal to use it in?

Four questions assessed various aspects of how panelists imagined VR might be best used within counseling sessions. In the first survey round, panelists agreed that while patients are using VR (Question 4), therapists should be flexible and decide what to do in the moment, which was somewhat incompatible exclusive with most potential items for this question that did not achieve consensus (e.g., “observe quietly while in VR, then debrief”). However, the one other item that achieved consensus for this question in the same round, “coach patients in real time,” was not incompatible with maintaining flexibility overall. Regarding what therapists should do *after* patients use a VR experience (Question 5), panelists agreed that therapists should do a number of basic things, including elicit patients’ general response to VR, review the choices they made in VR, inquire about what they learned, prompt them to reflect on applications of what they learned to their own lives, and explicitly link what they did in VR to their goals related to reducing or stopping use. However, similar to their advice for therapists while patients are in VR, panelists agreed that therapists should also be flexible in terms of processing VR and decide what to do in the moment based on patients’ needs, suggesting that they believed many of these steps should be adaptable. Most panelists agreed in the first rating round (Round 2) that the middle of a session would be most ideal for them to introduce and use VR with patients (Question 6). This question was dropped from subsequent rounds, since the remaining items were mutually exclusive. For Question 7, panelists agreed in the first rating round (Round 2) that, when introducing VR to patients, therapists should provide a general rationale for VR, emphasize VR’s utility as a practice or rehearsal tool, assess patients’ perspectives on whether and how VR might help, and inform them about potential adverse effects. Panelists also agreed that counselors could describe VR’s value for helping patients with their goal, express confidence in it, present data on it, or have patients do a tutorial or trial with VR to get familiar with it, but these items did not achieve consensus until the last rating round (Round 4). Qualitative comments suggest that these ratings likely reflect panelists beliefs that therapists should generally provide whatever introduction they believe would be most useful for their patients, based on what they know of their needs, whether that is providing data or easing them into it. This perspective is captured well by participant #10’s comment that: “*I think a lot of this depends on the patient. For example, one patient might be data driven and want to know what evidence supports this approach. Another might be more focused on the therapeutic relationship and want to know what you (the clinician) thinks about it*.”

Question 8 assessed what treatment settings panelists believed would be optimal for VR use. Consensus was achieved in the first rating round (Round 2) that all assessed settings, residential, inpatient, and outpatient, would be appropriate for VR use. However, in this round, 90% of panelists agreed that outpatient settings were appropriate for VR use, versus 70% for inpatient and residential settings, suggesting that more panelists were more comfortable with VR being used in outpatient settings, compared to inpatient and residential, which tend to serve those who are earlier on in treatment and higher-acuity patients. This sentiment was captured well by participant #6: “*I believe all settings could be valuable. It comes down to execution and the therapist using it in a way that best serves an individual patient*.”

#### Questions 9 and 10: Barriers to implementing VR in addiction treatment settings

Experts also described and then rated potential barriers to using VR in clinical settings. To elicit a wide range of potential concerns, we asked separately about “clinical barriers,” which referred to barriers “related to the interaction between the patient, the clinician, and the technology,” and “implementation barriers,” which referred to the “structural, operational, and financial frictions that could hinder VR use.” In practice, participants raised barriers of either type in response to both questions in Round 1. Barriers that achieved high consensus in early rating rounds (i.e., Round 2) mostly focused on problems using and maintaining the technology, cost, and therapist reluctance to use it, either because of low confidence with technology, unwillingness to troubleshoot it, or perceiving that it was not a good fit for their patients. The time needed to train therapists, lack of a defined reimbursement pathway for VR-assisted care, organizations’ IT policies, and perceptions that VR is more of a luxury than a core part of treatment were all barriers that panelists agreed were of concern. Notably, panelists largely agreed that *client* reluctance to use VR was unlikely to be a significant barrier.

## Discussion

In this study, we recruited experts in CBT for AUD to participate in a panel intended to generate expert consensus about when, where, and how VRmight be used within treatments inspired by CBT. In general, the panel was enthusiastic about the task, and provided very clear guidance about these issues, ultimately achieving agreement on all but a few items. Key findings from the panel are discussed by topic area below.

The first question we addressed was about what experts viewed as the most promising applications of VR in CBT for AUD. The panel most enthusiastically endorsed VR as a tool for actively rehearsing how they might respond in situations that are high-risk for relapse, such as social and stressful situations, practicing drink refusal and assertiveness skills, identifying triggers, and understanding the impact of seemingly irrelevant decisions on later risk for relapse. Qualitative comments largely suggested that panelists were especially enthusiastic about this application because it seems like an optimal fit with the technology’s strengths: immersing patients in a situation or scenario that feels more real so they can practice in a more realistic setting. However, panelists also emphasized that, for experiences like these to be effective in helping these skills generalize to patients’ real lives, they must be capable of recreating high-risk situations with enough fidelity that the skills practiced transfer. If VR cannot reach that threshold, traditional role-plays could work *better*, since patients can be asked to imagine the specific details that make a situation difficult for them, such as the influence of a particular friend or a familiar smell. The challenge for developers is to find a balance between creating experiences that are general enough to apply across many patients, yet personal enough to feel real. This challenge could be one reason why, despite panelists’ clear enthusiasm about the application, only one VR experience has been developed to date that aims to support goals like these (Thaysen-Petersen et al., 2024), and it relied exclusively on non-interactive, 360-video.

Notably, panelists also agreed that using VR to expose patients to alcohol-related cues could have value, despite limited evidence for treatments that rely on in-person exposure, like cue exposure therapy (Conklin & Tiffany, 2002). However, it is possible that VR-facilitated treatments are more effective than in-person versions (Huang et al., 2025; J.-H. Lee et al., 2007; Zhang et al., 2023), but larger, more definitive studies are needed to confirm. Finally, panelists were not enthusiastic about using VR to help patients improve the accuracy of assessment or self-monitoring or for depicting the consequences of heavy drinking, likely because it is not clear what value immersing patients in VR would add that could support these techniques beyond existing methods. These findings are consistent with the idea that VR would add the most value to techniques in which providing a realistic sense of presence supports practice and skill transfer (Grabowski & Jankowski, 2015; Seymour et al., 2002), and less to primarily informational techniques.

Beyond applications of VR, other questions asked panelists about when, where, and how VR might best be used when supporting these techniques, including how therapists might best introduce the concept of VR to patients, what therapists should do while patients are interacting with it, and how therapists might best help patients process what they experienced after using VR. Panelists’ responses in Round 1 and items that achieved consensus throughout Rounds 2–4 provide a menu of strategies therapists can use at each stage, from introducing VR, to guiding patients while they use it, to processing the experience afterward. However, the clearest message was that therapists should stay flexible and decide what to do in the moment based on each patient’s needs. Given these findings, the strategies endorsed by the panelists at each stage might be best viewed as a guide to potential strategies, rather than a script. This concept is consistent with the principle of flexibility within fidelity, which suggests that therapists deliver evidence-based treatments most competently when they adapt core elements to each patient rather than rigidly following a manual (Kendall et al., 2008; Kendall & Frank, 2018). Panelists also agreed that VR could be appropriate across outpatient, inpatient, and residential settings. This broad endorsement contrasts somewhat with reasonable concerns, raised by past studies, that exposing patients to alcohol cues too early in treatment, before abstinence is well-established, might increase the risk of relapse (Rohsenow et al., 1994; Witteman et al., 2015). Although this concern is most relevant for VR experiences that are explicitly designed to facilitate cue exposure, it may be best to reserve any experience that has the appearance of potentially precipitating relapse for later in care, since counselors who perceive a risk are unlikely to use it and counselor confidence is essential to adoption. Some panelists’ qualitative comments reflected this idea, suggesting that VR may be the best fit for later in patients’ treatment, as they begin to anticipate discharge.

Finally, panelists weighed in on the barriers most likely to impede VR’s use. The barriers rated most important primarily involved therapists: therapist reluctance to use VR, low confidence and comfort with technology, training time, and willingness to troubleshoot when something goes wrong. In fact, low confidence with technology among therapists was the only barrier endorsed by every panelist in the first rating round. By contrast, the panel agreed that several patient-focused concerns were not important, including clients’ own confidence with technology and discomfort at being watched. This finding is consistent with ample past research showing that patients are enthusiastic about using VR in counseling sessions (Arissen et al., 2022; Garcia-Palacios et al., 2007; Levy et al., 2023), and, contrary to the common worry that therapists are reluctant, several studies show high interest among clinicians as well (Arissen et al., 2022; Segal et al., 2011; Wray & Emery, 2022). Future research should test whether this enthusiasm actually translates into sustained use, and what happens when therapists encounter practical frictions of using it with patients.

Ensuring that the technology works as smoothly as possible and with minimal maintenance was also among the top concerns raised by panelists. A headset that fails to connect or frequently needs updating interrupts the flow of sessions and risks undermining confidence that the panel viewed as essential to encouraging therapist uptake. Unlike other devices (e.g., computers, smartphones), few therapists are familiar with VR or use it themselves outside of work, so requiring them to troubleshoot problems with a complex and unfamiliar technology could quickly lead them to abandon it. Relatedly, the panel agreed that viewing VR as a luxury rather than an essential part of treatment is also likely to be an important barrier to adoption and long-term use. This concept, known in implementation frameworks as *relative advantage* (Damschroder et al., 2022), is especially important when there is an effective alternative that therapists regularly use (e.g., traditional role-plays). Experiencing technical problems mid-session becomes a much bigger deterrent to using VR when therapists can simply revert to the traditional approach, unless there is clear evidence that VR provides benefit beyond the alternative. Designers should therefore ensure that VR systems work as smoothly and reliably as possible, that any problems can be resolved quickly, and that technical support is easy to reach. Evidence of VR’s added benefit over established techniques is also essential.

This study had several important strengths, including an especially accomplished panel of experts, their dedication to the task, and complete retention through the final survey round. However, limitations are also important to note. First, although there is no firm standard for panel size in Delphi studies (Akins et al., 2005), this panel was small (*N* =10). A larger panel might have had more disagreement across items. Second, all panelists were doctoral-level psychologists based in the United States, and all identified as White, which limits the representation of frontline counselors, other disciplines, international and racial/ethnic diversity. Third, ratings were based on hypothetical VR use rather than direct clinical experience, and expert opinion may be subject to desirability and other biases. Finally, consensus reflects what experts believe is most promising, which may not align with what actually improves patient outcomes. Future research should evaluate this approach to determine clinical benefit.

In summary, the results of this study provide a clear, expert-informed blueprint for how a VR tool could support CBT-inspired treatments for AUD. The characteristics of this blueprint are summarized in Table 3. These findings can directly guide the design of such platforms, specifying where VR is most likely to augment established techniques and how it should be used. Future research should test whether such a platform actually improves patient outcomes in rigorous, well-controlled trials.

## Figures and Tables

**TABLE 1.** *Characteristics of the expert panel (N* = 10)

Characteristic	*N* (%) or *M* (*SD*)
Age	50.4 (9.7)
*Gender*	
Female	3 (30.0)
Male	7 (70.0)
Doctorate degree	10 (100.0)
Years since earning doctorate	22.6 (12.4)
*Professional title*	
Assistant professor	2 (20.0)
Associate professor	2 (20.0)
Professor	6 (60.0)
h-index^1^	41.8 (18.7)
*Employment status*	
Full-time	8 (80.0)
Part-time	1 (10.0)
Retired	1 (10.0)
Race	
White	10 (100.0)
Black	0 (0.0)
Native American/Alaska Native	0 (0.0)
Asian	0 (0.0)
Pacific Islander	0 (0.0)
Multiracial	0 (0.0)
Hispanic/Latino	1 (10.0)

Note.

1 As recorded from Google Scholar and Scopus. We collected h-index to characterize the level of scholarly expertise of the panel.

**TABLE 2.** Summary of items that were endorsed, not endorsed, and that achieved no consensus by the final round

Question	Consensus reached (>70% agreement)		Consensus not achieved
	Endorsed^1^	Not endorsed^1^	
Q1: What techniques, skills, or exercises would benefit most from incorporating VR?	Practicing drink refusal skills and assertive communication Helping patients learn and practice managing high-risk situations Exposing patients to alcohol cues in high-risk contexts Identifying potential triggers Simulating scenarios where seemingly irrelevant decisions increase relapse risk Anticipating future high-risk situations Relaxation training	Improving the accuracy of assessment Illustrating or simulating the consequences of alcohol use or intoxication Experiencing reinforcement for verbalizing a commitment to change Helping patients self-monitor and log feelings, thoughts and cravings Simulating a relapse experience to create a relapse prevention plan Simulating giving or receiving social support	Identifying or simulating substance-free alternative activities
Q2: What kinds of VR environments would be most helpful?	Social situations Stressful situations	--	--
Q3: What elements within these VR environments would be most impactful for a clinician to be able to control, modify, or customize?	Type of common drinking environment Level of difficulty or intensity of a situation Types of alcohol depicted Age of other people in scenario Race or ethnicity of other people in scenario Other people are known to the patient Sex or gender of the other people Amount of other people Degree of unpredictability in events that occur	Music or noise level	--
Q4: What should therapist’s role be *while* patients are in VR?	Coach patients in real time Be flexible and decide what to do in the moment based on patient needs	--	--
Q5: What should therapists do *after* patients finish using VR to maximize its impact?	Elicit patients’ general response to VR Inquire about what patients learned Review the choices made in VR Affirm appropriate use of skills Prompt patients to reflect on what they might do in similar real-world situations Explicitly link responses to goals related to reducing or stopping use Re-assess important metrics Avoid conveying to patients that there are right or wrong answers Be flexible and decide what to do in the moment based on patient needs	--	Complete the form corresponding to the VR activity to promote between-session skill use
Q6: When during a session should VR be introduced?	Middle	--	--
Q7: How should VR be introduced to patients in session?	Provide a general rationale for VR Emphasize VR’s utility as a practice or rehearsal tool Describe VR’s value for helping patients with their goals Assess patients’ perspectives on whether and how VR might be useful to them Express confidence in VR as a helpful treatment tool Inform patients about potential adverse effects	Present data on the efficacy of VR-assisted counseling Encourage a brief, non-therapeutic VR trial to familiarize patients	
Q8: What treatment settings would VR be an optimal fit for?	Residential Inpatient Outpatient	--	--
Q9: What might be the most important *clinical* barriers to using VR in AUD treatment?	Technology failing to work or breaking Maintaining the software Maintaining the headsets and hardware Therapist reluctance to use VR Therapist concerns that VR is not a good fit for their patients Therapist concerns that patients might leave the session triggered or stressed Low technology literacy, fear, or distrust of technology among therapists VR experiences are not realistic enough depictions of patients’ real experiences Patient fears about experiencing something scary or intense in VR Patients will not want to use VR Low technology literacy, fear, or distrust of technology among patients	Using VR could harm the therapeutic alliance Not using a common or popular VR system VR could take up too much time in sessions Unclear how therapists could provide feedback for home VR use Insufficient number of relevant VR experiences available Patient discomfort with being watched or unable to see their real environment while using VR	Conveying VR as mirroring real experiences rather than “mock” practice situations Patients could get motion sickness or have other adverse reactions
Q10: What might be the most important *implementation* barriers to using VR in AUD treatment?	Cost Therapist willingness to troubleshoot technology problems Access to sufficient technical support Therapist training time Therapist perceptions that VR is a luxury rather than a core component of treatment Lack of a defined reimbursement pathway for VR-assisted care Organizations’ IT policies and processes Relative importance of VR versus other techniques	Space limitations Client confidence or self-efficacy with technology	Limited evidence of clinical benefit or efficacy so far

Note.

1 Endorsed refers to the required ≥70% of panelists agreeing that the item is important. Not endorsed refers to the required ≥70% of panelists agreeing that the item is *not* important. *Consensus required ≥70% of panelists rating an item 4–5 (endorsed) or 1–2 (not endorsed)*.

**TABLE 3.** Expert-endorsed profile of VR tools to support CBT for AUD

Domain	What experts endorsed
**Best applications**	Rehearsing responses to high-risk situations, including drink-refusal and assertiveness skills, alcohol cue exposure, trigger identification, seemingly irrelevant decisions, and relaxation training.
**Experience types**	Realistic social and stressful situations.
**Customization**	As many aspects as possible: drinking environment, control over cue exposure, difficulty or intensity, alcohol type, and the characteristics, number, and unpredictability of others
**Introducing VR**	Frame VR as a rehearsal tool tied to the patient’s goals; give a rationale, disclose possible adverse effects, assess the patient’s perspective, and convey confidence.
**Therapist role during use**	Coach in real time, stay flexible and decide based on patient needs
**Therapist role after use**	A structured debrief: affirm skills, prompt real-world reflection, link to goals, ask what was learned and whether it felt real, review choices, re-assess craving and anxiety, and avoid right/wrong framing.
**Timing and setting**	Use in the middle of a session; appropriate for outpatient, inpatient, and residential care.
**Key adoption barriers**	Therapist-facing (reluctance, low technology confidence, training time) and structural (cost, technical support, reimbursement, IT policy).

## Data Availability

Item-level Delphi results and surveys are available from the corresponding author on reasonable request.
